# A descriptive study of allergen-specific IgE serological tests for canine atopic dermatitis in Thailand

**DOI:** 10.1186/s12917-020-02684-x

**Published:** 2020-12-07

**Authors:** Suttiwee Chermprapai, Naris Thengchaisri

**Affiliations:** 1 Department of Companion Animal Clinical Sciences, Faculty of Veterinary Medicine, Kasetsart University, 10900 Bangkok, Thailand; 2 Dermatology Unit, Kasetsart University Veterinary Teaching Hospital, 10900 Bangkok, Thailand

**Keywords:** Allergy, Allergen-specific IgE test, Atopic dermatitis, Dogs, House dust mite

## Abstract

**Background:**

This study describes the usefulness of allergen-specific Immunoglobulin E (IgE) serology (ASIS) for identifying allergens in dogs with atopic dermatitis. ASIS tests were conducted in 23 dogs diagnosed with atopic dermatitis for indoor allergens (yeast and mites), outdoor allergens (grass pollen, weed pollen, and tree pollen), and fleas. The relationship among positive ASIS tests were determined using Pearson’s correlation coefficient (r).

**Results:**

Of the atopic dogs, 26.09%, 4.35%, and 47.83% had positive ASIS tests for only indoor allergens, only outdoor allergens, and both indoor and outdoor allergens, respectively. The prevalence of positive ASIS tests was highest for mites (69.57%) and did not differ between indoor and outdoor allergens by age, breed, or sex. The prevalence of positive ASIS tests for indoor allergens during the rainy season (84.21%) was significantly higher than during winter (25.00%, *P*-value = 0.030). The correlation coefficient of the ASIS results among the outdoor allergens indicated a strong correlation between grass and tree pollen (r = 0.840, *P*-value < 0.01), grass and weed pollen (r = 0.812, *P*-value < 0.01), and tree and weed pollen (r = 0.714, *P*-value < 0.01). The correlation coefficient of the ASIS results of *D. farinae* indicated a strong correlation with *A. siro* (r = 0.951, *P*-value < 0.01) and a moderate correlation with *B. tropicalis* (r = 0.656, *P*-value < 0.01) and *T. putrescentie* (r = 0.672, *P*-value < 0.01).

**Conclusions:**

ASIS tests are useful in screening for multiple allergens in dogs with atopic dermatitis. Dust mites are an important source of indoor allergens and may be responsible for a higher titer of IgE antibodies against indoor allergens during the rainy season.

## Background

Canine atopic dermatitis (CAD) is a chronic multifactorial inflammatory and pruritic allergic skin disease with distinctive clinical features [1]. The pathogenesis of CAD has been linked to skin barrier dysfunction, immune responses to indoor and outdoor allergens, and complicated infections [2–4]. Elevation of serum Immunoglobulin E (IgE) is commonly found in CAD and is believed to be one of the main mediators for hypersensitivity reactions [5]. The recent development of allergen-specific IgE serology (ASIS) have contributed to the rapid identification of common indoor/outdoor allergens among dogs with atopic dermatitis (AD) [6]. The usefulness of ASIS tests in clinical settings compared with intradermal skin tests (IDTs) for specifying the causal allergens remains controversial [7]. IDTs are commonly used in clinical practice, even though the testing may lead to patient discomfort as well as anaphylactic reactions [5]. ASIS tests cause less pain for canine patients without the need to shave the dog’s hair and use sedative agents [7]. The application of ASIS tests in atopic dogs provides a good solution for conditioned patients.

Different types of allergens can be synchronously investigated within the same test [8]. The most common allergens in CAD are house dust mites, storage mites, pollen, molds, and epidermal allergens; the allergens involved can vary by geographic location [8]. Selecting relevant allergens in a patient’s local region helps increase the chance of finding significant test results [7]. Research investigating the usefulness of allergen identification in atopic dogs in Thailand is limited and has been performed only by IDT not ASIS [9]. Previous studies reported the influence of breed on atopic dogs and links to possible genetic-related problems [10]. Findings on the influence of age, body size, and sex among these dogs have been inconsistent [11–13]. It is possible that seasonal influence may also affect the results of ASIS tests [11]. Thailand is located in a tropical region, Southeast Asia, and has three seasons:  summer, rainy season, and winter; which differs from temperate and subpolar regions that have four seasons: summer, spring, autumn, and winter [14, 15]. Most earlier studies observed that dogs with AD tend to develop clinically allergic responses in the spring and summer [16–18].

The objective of the present study was to evaluate the usefulness of ASIS tests for identifying specific indoor and outdoor allergens in atopic dogs. The relationship between positive ASIS results among different allergens, as well as the influence of climate conditions, was compared.

## Results

**Fig. 1:**
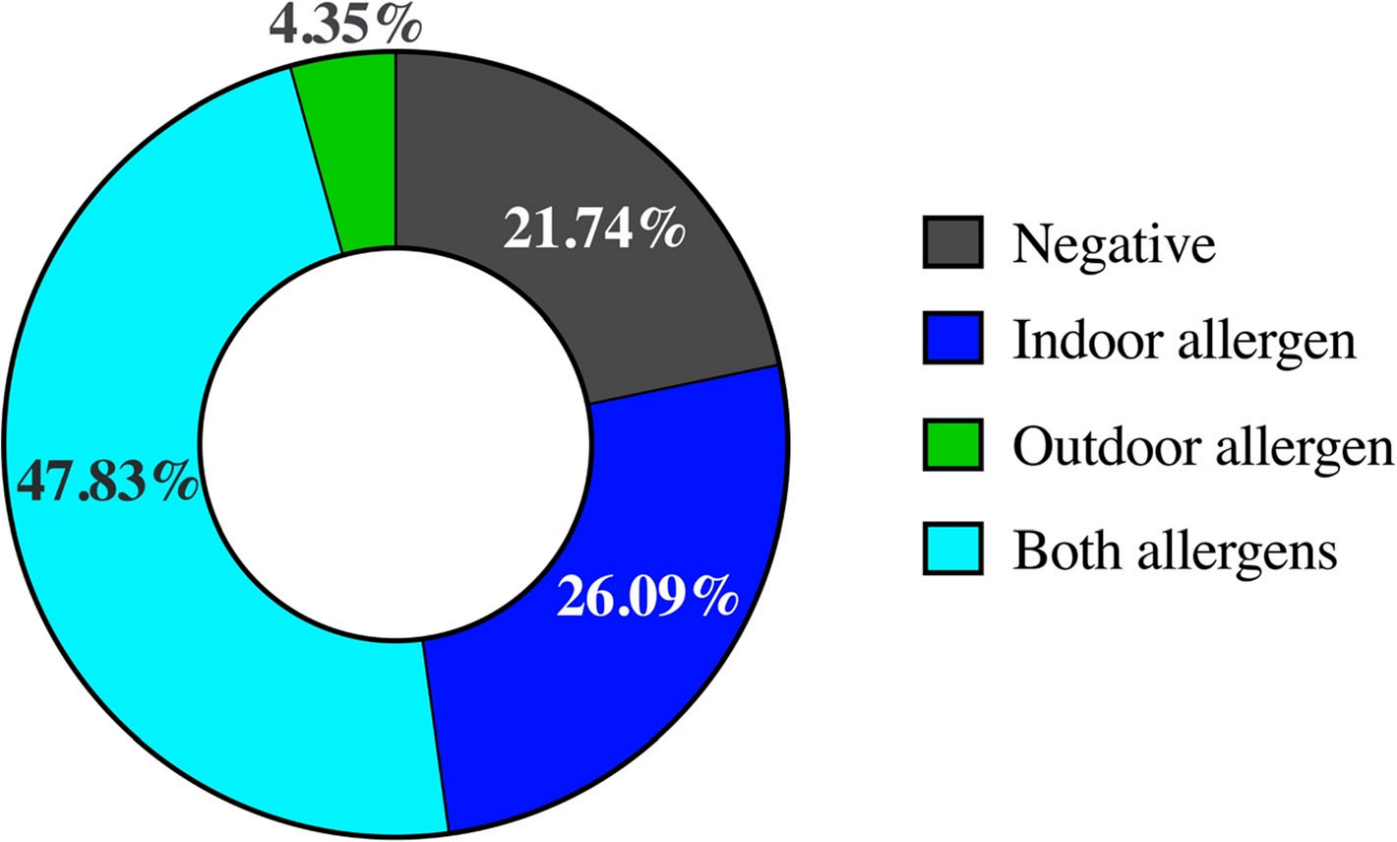
Summarized results of allergen-specific IgE serological (ASIS) tests in atopic dogs classified by indoor and outdoor allergens

The percentage of atopic dogs with ASIS tests positive for only indoor allergens (yeast and mites), only outdoor allergens (grass pollen, weed pollen, and tree pollen), and both indoor and outdoor allergens was 26.09%, 4.35%, and 47.83%, respectively (Fig. 1). Of the atopic dogs, 21.74% had negative ASIS test results. The prevalence of ASIS tests positive for specific allergens from highest to lowest was as follows: mites (69.57%), weed pollen (43.48%), grass pollen (34.78%), tree pollen (30.43%), yeast (30.43%), and fleas (0.00%). The prevalence of positive ASIS results for the allergens examined is shown in Table 1. Among indoor allergens, the prevalence of house dust mites (*D. farinae* = 56.52%) and storage mites (*T. prutescentie* = 56.52%; *A. siro* = 52.17%; *B*. *tropicalis* = 30.43%) was found to be highest. Among outdoor allergens, grass pollen was most prevalent (17.39–30.43%). The prevalence of positive ASIS tests for indoor and outdoor allergens in atopic dogs did not differ according to age, breed, and sex (Table 2). The number and percentage of atopic dogs with a positive or negative ASIS score for the 6 types of allergens are provided in Table 3. These results indicated that mite and yeast were the predominant indoor allergens, respectively. Weed pollen, tree pollen and grass pollen were the most dominant outdoor allergens, respectively. The share of positive ASIS tests among atopic dogs for indoor allergens during the rainy season (84.21%) was significantly higher than that found during the winter season (25.00%, *P*-value = 0.030; Table 4). The share of positive ASIS tests for outdoor allergens during the rainy season (52.63%) was comparable to that found during the winter season (50.00%, *P*-value = 0.127; Table 4).

**Table 1:** Percentage and number of atopic dogs with positive results to different allergens according to the allergen-specific IgE serological (ASIS) tests

Number	Name of allergen	Group	Number of positive IgE (n = 23)	Percentage of positive IgE
1	*Malassezia pachydermatis*	1	7	30.43
2	Flea saliva	2	0	0.00
3	*Dermatophagoides farinae*	3	13	56.52
4	*Blomia tropicalis*	3	7	30.43
5	*Tyrophagus putrescentiae*	4	13	56.52
6	*Acarus siro*	4	12	52.17
7	Bermuda grass (*Cynodon dactylon)*	5	5	21.74
8	Bahia grass (*Paspalum notatum*)	5	4	17.39
9	Johnson grass (*Sorghum halepense*)	5	4	17.39
10	Meadow fescue (*Festuca pratensis*)	5	7	30.43
11	Timothy grass (*Phleum pratense*)	5	5	21.74
12	Red/sheep sorrel (*Rumex acetosella*)	5	6	26.09
13	Lamb’s quarter (*Chenopodium album*)	5	4	17.39
14	Russian thistle (*Salsola kali*)	6	5	21.74
15	Careless weed (*Amaranthus hybridus*)	6	5	21.74
16	Cocklebur (*Xanthium strumarium*)	6	3	13.04
17	Marsh elder (*Cyclachaena xanthiifolia*)	6	3	13.04
18	Common and giant ragweed (*Ambrosia artemisiifolia* and *A. trifida*)	6	5	21.74
19	English plantain (*Plantago lanceolate*)	6	5	21.74
20	Mugwort (*Artemisia vulgaris*)	6	2	8.70
21	Paperbark tree (*Maleleuca quinquenervia*)	7	2	8.70
22	White mulberry (*Morus alba*)	7	4	17.39
23	Queen and oil palms (*Arecastrum romanzoffianum* and *Elaeis* *guineensis*)	7	5	21.74
24	White oak (*Quercus alba*)	7	3	13.04

Group category 1: Yeast, 2: Flea, 3: House dust mite, 4: Storage mite, 5: Grass pollen, 6: Weed pollen, 7: Tree pollen

**Table 2:** Allergen-specific IgE serological (ASIS) test results in atopic dogs by age, sex, and breed

Category	Subtype	No. positive	No. negative	% positive	*P*-value
**Indoor IgE allergens**					
Age group	Young (1–3 years)	7	1	87.50	—
	Adult (> 3 years)	10	5	67.67	0.369
Breed	Small	4	2	67.67	—
	Medium	8	3	72.73	1.000
	Large	5	1	83.33	1.000
Sex	Male	7	4	63.64	—
	Female	10	2	83.33	0.371
**Outdoor IgE allergens**					
Age group	Young (1–3 years)	5	3	62.50	—
	Adult (> 3 years)	7	8	46.67	0.667
Breed	Small	2	4	33.33	—
	Medium	8	3	72.73	0.162
	Large	2	4	33.33	1.000
Sex	Male	4	7	36.36	—
	Female	8	4	66.67	0.220

**Table 3:** Percentage and number of atopic dogs according to allergen specific IgE serological (ASIS) scores among different groups of allergens (yeast, mites, grass pollen, weed pollen, and tree pollen)

Allergy group	No. of dogs with IgE score of 2–4	No. of dogs with IgE score of 0–1	% positive	*P*-value
Yeast	7	16	30.43	—
Flea	0	23	0.00	0.004
Mite	16	7	69.57	0.008
Grass pollen	8	15	34.78	0.753
Weed pollen	10	13	43.48	0.360
Tree pollen	7	16	30.43	1.000
Overall	18	5	78.26	0.003

**Table 4:** Seasonal effects on the results of allergen-specific IgE serological (ASIS) tests in atopic dogs

Category	Season	No. positive	No. negative	% positive	*P*-value
Indoor allergens	Rainy	16	3	84.21	—
	Winter	1	3	25.00	0.030
Outdoor allergens	Rainy	10	9	52.63	—
	Winter	2	2	50.00	0.127
Both indoor and outdoor allergens	Rainy	16	3	84.21	—
	Winter	2	2	50.00	0.064

The correlation coefficient of the ASIS results among the outdoor allergens indicated a strong correlation between grass pollen and tree pollen (r = 0.840, *P*-value < 0.01), grass pollen and weed pollen (r = 0.812, *P*-value < 0.01), and tree pollen and weed pollen (r = 0.714, *P*-value < 0.01; Table 5). The correlation coefficient of the ASIS results of *D. farinae* (Table 5) indicated a strong correlation with *A. siro* (r = 0.951, *P*-value < 0.01) and a moderate correlation with *B. tropicalis* (r = 0.656, *P*-value < 0.01) and *T. putrescentie* (r = 0.672, *P*-value < 0.01).

**Table 5:** Pearson’s correlation coefficient (r) among groups of indoor allergen (*A. siro, B. tropicalis, D. farinae, T. putrescentie*) or outdoor allergens (yeast, mites, grass pollen, weed pollen, and tree pollen) in atopic dogs based on allergen-specific IgE serological (ASIS) tests

**Indoor IgE allergens**				
Mite allergen	*Acarus siro*	*Blomia tropicalis*	*D. farinae*	
*Tyrophagus putrescentie*	0.789**	0.616**	0.672**	
*Acarus siro*	—	0.716**	0.951**	
*Blomia tropicalis*	—	—	0.656**	
**Outdoor IgE allergens**				
Allergen group	Grass pollen	Weed pollen	Tree pollen	Yeast
Mite	0.288	0.305	0.288	0.308
Grass pollen	—	0.812**	0.840**	0.205
Weed pollen	—	—	0.714**	0.346
Tree pollen	—	—	—	0.140

** Indicates a *P*-value of <0.01

## Discussion

In the present study, ASIS tests were performed for dogs with atopic dermatitis across 24 allergens. Our findings indicate the important role of indoor allergens over outdoor allergens. House dust mites and storage mites were the predominant allergens among the atopic dogs. We also identified that during the raining season, indoor allergens were the main source of allergens. It should be noted that the positive ASIS results of the outdoor allergens (grass pollen, weed pollen, and tree pollen) were highly correlated. Positive ASIS results for house dust mite allergens were moderately to highly associated with positive ASIS results for storage mites.

Unlike human medicine, for which several atopic dermatitis-related genes have been studied [19, 20], only the gene related to IgE production has received attention in veterinary medicine, and a limited correlation has been observed between clinical atopic dermatitis development and IgE levels or other genes [21–24]. The updated practical guidelines to diagnose CAD include ruling out other pruritic skin diseases, interpreting the clinical features as described as Favrot’s criteria, and identifying causal allergens either through IDTs or ASIS testing [7]. Although ASIS can have poorer specificity and lower positive predictive value compared with IDTs, ASIS tests provide several advantages over IDTs, such as (1) no patient risk related to sedation or of anaphylactic reactions, (2) convenience (no hair clipping, no restraint), (3) a lower likelihood of influence related to prior or current drug therapy, and (4) shorter duration (easily done in a minute) [25]. Identifying allergens by ASIS testing is favorable when patients have certain limitations that preclude the use of an IDTs, like most of the patients enrolled in this study. In the current study, there were some dogs with clinical features of AD but an undetectable IgE response to allergens; this can be explained by the possible confounding problems of recruiting patients with atopic-like dermatitis or by the fact that the ASIS testing cannot detect the presence of cutaneous mast cells with reaginic antibodies present on the mast cells [25, 26].

It has been described previously that ASIS tests measured by enzyme-linked immunosorbent assay (ELISA) can be analyzed by using mono- or polyclonal antibodies or the high-affinity Fc epsilon receptor alpha chain protein (FcεRIα), and positive results have varied among these assays [27, 28]. Although the chance of cross-reactivity between IgE and IgG has been discussed [29], the serum samples of this study were analyzed by the patented Fc-ε receptor testing method (Allercept), which can provide a low chance of cross-reactivity of positive results [30]. However, the cross-reactivity in the same group of allergens, such as house dust and storage mites, remains and cannot be ruled out [31]. Another study demonstrated that the prevalence of positive ASIS results using the high-affinity FcεRIα was similar or even better than the results of IDTs [32]. It is noteworthy that the correlation between the results of ASIS tests and IDTs possibly ranged from weak to strong depending on numerous factors, including standardization of allergens, testing technique and laboratory conditions, or individual factors [33–35]. Positive results of IDTs and ASIS tests should be compared with caution. The proper selection of diagnostic tools helps improve the sensitivity and the specificity of the test and thus the ability to reliably identify patients’ clinically relevant allergens. Performing both an IDTs and ASIS testing can provide better outcomes with which to interpret an allergic response to tested allergens [36].

The clinical signs of CAD vary depending on several factors, including age of onset, breed, sex, anatomical site, type of skin lesion, and seasonality [11]. Seasonality varies with respect to geographic location and relates to environmental allergens [11]. Spring and summer have been most commonly described as related to the development and progression of allergic responses [16, 17]. Thailand is located in Southeast Asia and has three seasons: summer, the rainy season, and winter [15]. In our study, most of the patients’ serum samples were taken in the rainy and winter seasons with regard to the clinical presentation. It was found that during the rainy season, indoor allergens were the main source of the allergens compared with the outdoor allergens. House dust mites, the major indoor allergen related to atopic dermatitis, prefer tropical and subtropical climates with relatively high humidity [37, 38]. When rainfall is heavy during the rainy season in Thailand, owners likely keep their animals indoors more often, which may lead to increased exposure to house dust mites. The effect of seasonality on CAD development should be further studied and include all the seasons involved in the studied region.

The limitations of the present study include the cross-reactivity, the seasonality effects, and the downsides of ASIS testing mentioned earlier, as well as the sample size. No significant differences in positive ASIS results were observed for indoor and outdoor allergens based on age, breed, or sex. Given the retrospective nature of the present study, we did not have cases with flare-up symptoms in summer during the period of the current study for ASIS testing. More studies can be performed to identify the possible risks associated with the raining season resulting in the flare-up symptoms of the atopic dogs. It is possible that the sample size used in the present study may be limited for identifying difference in positive ASIS based on age, breed, or sex. Although none of the atopic dogs enrolled in this study had positive ASIS results for flea antigens, this finding is fairly in agreement with earlier studies that found only a low prevalence of positive results for flea antigens [12, 39]. Improved population sampling and/or increased sample size of the study may have provided a more rigid conclusion about serology testing for dermatitis related to flea allergies as well as multivariate analysis of the factors associated with CAD.

## Conclusions

Multiallergen screening using ASIS tests revealed that house dust mites and weed pollen are the most important sources of allergens in Thailand. The present findings indicated that during the rainy season, atopic dogs may experience a higher titer of IgE antibodies against indoor allergens. Cross-reactivities from ASIS tests likely occurred among dust mite allergens as well as different types of pollen. ASIS testing serves the purpose of identifying allergens that may be included in the treatment. The application of ASIS tests in atopic dogs provides a good solution for conditioned patients.

## Methods

### Animals

This study was approved by the Kasetsart University Institutional Animal Care and Use Committee (ACKU63-VET-044) and by the Ethical Review Board of the Office of National Research Council of Thailand (NRCT license No. U1-08491-2562). Twenty-three client-owned dogs with atopic dermatitis (12 females and 11 males, median age of 4.3 ± 4.0 years) of different breeds that visited the Dermatology Unit at Kasetsart University Veterinary Teaching Hospital from 2017 to 2019 were included. The dog owners were informed and educated about the practical approach of pruritic problem. The dogs met Favrot’s diagnostic criteria for atopic dermatitis, and other pruritic skin diseases were ruled out by direct examination of the ectoparasites, coat brushing, skin scrapings, and regular use of antiparasitic control [7]. Infection and inflammation caused by bacteria, fungi, and/or yeast were ruled out by skin cytology. Adverse food reaction was ruled out by an elimination diet trial of 8–12 weeks. No concurrent anti-inflammatory, antihistamine or antibacterial/antifungal treatments were allowed during the elimination diet trial or 4 weeks prior to serum collection for ASIS.

### Allergen-specific IgE serology (ASIS)

At least 5 mL of serum sample was collected from each dog by cephalic venipuncture and dogs were returned to the owner after blood collections. All serum samples were submitted to Animal Allergy Lab, Heska’s International Lab Partners in Singapore, for measurement of allergen-specific IgE (Allercept® allergen specific IgE test, Heska Corp., Singapore). The Asian panel was categorized into 7 groups, including indoor and outdoor allergens and fleas (Table 1). The indoor allergens included were house dust mites (*Dermatophagoides farinae* and *Blomia tropicalis*), storage mites (*Tyrophagus putrescentiae*), and flour mites (*Acarus siro*). The outdoor allergens included were Bermuda grass (*Cynodon dactylon*), Bahia grass (*Paspalum notatum*), Johnson grass (*Sorghum halepense*), meadow fescue (*Festuca pratensis*), Timothy grass (*Phleum pratense*), red/sheep sorrel (*Rumex acetosella*), lamb’s quarter (*Chenopodium album*), Russian thistle (*Salsola kali*), careless weed (*Amaranthus hybridus*), cocklebur (*Xanthium strumarium*), marsh elder (*Cyclachaena xanthiifolia*), common and giant ragweed (*Ambrosia artemisiifolia* and *A. trifida*), English plantain (*Plantago lanceolata*), mugwort (*Artemisia vulgaris*), paperbark tree (*Maleleuca quinquenervia*), white mulberry (*Morus alba*), queen and oil palms (*Arecastrum romanzoffianum* and *Elaeis guineensis*), and white oak (*Quercus alba*). Yeast (*Malassezia pachydermatis*) and fleas (flea saliva), commonly related to CAD, were also included in the analysis. For further information regarding the identification of plant materials as well as the acquisition of allergens, please visit the company website (www.heska.com). Based on the concentrations of IgE measured, results were stratified into one of four classes: 0 = no reaction, 1 = a light nonspecific reaction, 2 = a weak positive reaction, 3 = a moderate positive reaction, and 4 = a strong positive reaction. Reactions of greater than or equal to 2 were considered positive ASIS test results.

### Statistical analysis

The sample size was calculated using freely downloadable software G*Power3.1 (Faul, Erdfelder, Lang and Buchner, 2007) to detect a difference using an exact test with alpha = 0.05 (two-tailed test), beta = 0.9, and an effect size of 0.25. The data were analyzed using STATA12 (StataCorp, College Station, Texas, USA) and GraphPad Prism Version 6 (GraphPad Software, San Diego, California, USA). The prevalence of ASIS results for different allergens, as well as the prevalence of ASIS results acquired during the rainy season and winter, was compared using Fisher’s exact test. The relationship among ASIS results for different allergens was determined using Pearson’s correlation coefficient (r). A *P*-value < 0.05 was considered statistically significant.

## Data Availability

The data used and/or analyzed in the present study are available from the corresponding author on reasonable request.

## Abbreviations

AD: Atopic dermatitis
ASIS: Allergen-specific IgE serology
CAD: Canine atopic dermatitis
IDT: Intradermal skin tests
IgE: Immunoglobulin E
